# The Comic History of British Nursing

**Published:** 1913-05-31

**Authors:** Adelaide Nutting

**Affiliations:** Teachers College, Columbia University, New York.


*To the Editor of* The Hospital.

### The Comic History of British Nursing.

*To the Editor of* The Hospital.

Sir,—My attention has been called to a review of the "History of Nursing," Volumes III. and IV., published in your issue of March 8. This review appears to me to be not only markedly unfair and misleading, but to have been written in a deeply hostile spirit, and solely for the purpose of discrediting the "History." I therefore deem it my duty to try to correct as far as I am able some at least of the mistaken impressions which the review might leave in the minds of your readers.

Your reviewer intimates that the history has been written by a "small clique" . . . who have collaborated in "mutual laudation," and in "depreciation of all opponents." He calls it a "farrago of prejudice masquerading as history," and adds the grave accusation that its "misrepresentations of facts alone are sufficient to condemn it." Let me reply in the interests of truth and accuracy that the history has not been written, as your reviewer intimates, by a "small clique," but by a very large number of women in various countries, nurses who have helped to make the history of nursing, and can speak from an intimate and full knowledge of the work and events in which they have participated.

To whom, pray, would you turn for accurate information concerning nursing but to those nurses whose ability and devotion to their calling have led them to study its problems for the purpose of improving it and rendering it more efficient? And so far from being "animated by prejudice," the attitude of the writers from various countries seems on the whole moderate and restrained. Doubtless unpalatable truths are presented. It would be difficult indeed to write any true History of Nursing during the past quarter of a century, at least, which would form pleasant reading for those who, in hospitals or out of them, have been concerned with that enterprise which we can only truthfully call the exploitation of nurses. The historian of the future who will have access to the facts will probably have to present a still less pleasant picture. In questioning the veracity of the various authors, your reviewer should be specific in his statements, and thus enable the authors to reply to him.

As my name appears in the review, the opportunity is given which I gladly use, to state that I believe Volumes III. and IV. are history in a much truer sense than the first two volumes, since in the latter access has been had to first-hand sources. And in this connection let me add that my share in the production of these first volumes is entirely insignificant compared with that contributed by Miss Dock. The history would never have appeared at all had it not been for the generous and liberal way in which Miss Dock took hold of the plan and worked it out, devoting her full time for nearly two years to the task, and bringing to it a great amount of careful research and study, and that freshness, spontaneity, and originality which has characterised all of her writings. There is no literary value in the book except that which has been contributed by Miss Dock.

Let me further say that I would have highly valued the honour of being associated with Miss Dock in the production of the last two volumes. The idea of these volumes is entirely hers, the work in securing, arranging, and editing the material is hers; that of preparing it for publication is all hers; and the proceeds which come from these books she has, with characteristic generosity, presented to the International Council of Nurses. To many of us the only real defect of the last volumes is the omission of any reference whatever to the large, important, and uniquely valuable share which Miss Dock has had in the development of nursing in this country. It is doubtful, to my mind, if any one of our number has rendered greater service than she has rendered, and for the benefit of future generations of nurses this lack in the history should in some way be supplied.

I shall be indebted to you if you will kindly publish this in an early issue of your Journal, and would say that I am also sending a copy to the *British Journal of Nursing*.—Believe me, faithfully yours,

Adelaide Nutting.

Teachers College, Columbia University, New York.
May 7, 1913.

* [Miss Nutting should read again, and more carefully, the review of which she complains. It was *not* stated, as she alleges, that the "History of Nursing" was written by a small clique; but only that the first section of its third volume was thus constructed. It will, therefore, be seen that the greater part of Miss Nutting's second and third paragraphs fall to the ground, because they are based on a misconception so gross that we cannot understand how she ever fell into it. As for the "mutual laudation," "prejudice," and so forth, that again was only attributed in our review to the first section of the third volume. We do not retract a word of it, and anyone who reads those chapters can judge of it for himself.

"To whom, pray," says Miss Nutting, "would you turn for accurate information concerning nursing but to those nurses whose ability and devotion to their calling have led them to study its problems. . . . " This sentence ignores deliberately the whole gist of our complaint; but if the point was not made plain in the review, let us try to present it more clearly to Miss Nutting and to whomsoever else it may concern. A controversy arose in Great Britain some years ago over the question

(*Continued on page* 292).

whether or not State registration of nurses was desirable. Some nurses and other individuals held that it was, and advocated that view with great persistence and energy. Other nurses and individuals held the opposite view; and so far their arguments have prevailed. In other words, the British upholders of State registration have been the defeated party. Miss Dock's history contains a section on the history of British nursing, contributed entirely by the leaders of that party. Their contribution consists of the most fulsome mutual admiration, the execrable taste of which Miss Nutting, we note, does not attempt to defend; and of fierce denunciation of the victorious party. We remarked on Miss Dock's way of compiling history, and apparently Miss Nutting sees nothing unusual about it.

It will easily be understood that "history" thus compiled is hardly likely to be impartial; and the whole gravamen of our complaint was that partiality and prejudice were in evidence throughout this particular section of Miss Dock's book. That is a matter which anyone can test for himself by referring to the book itself. We are not afraid of the application of this test by anyone who wants to satisfy himself about the matter. Such an inquirer will find that the section on British nursing in Volume III. of Miss Dock's book is composed by four or five collaborators, whose photographs (with one exception) occupy prominent places in the book: that the section extols the ways and works of this clique in language of adulation and exaggeration; and that the most despicable motives are freely attributed to all who differ from this band of mutual admirers. If that does not satisfy him that our review gives an accurate statement of the case, all we can say is that standards of good taste and the amenities of fair controversy have deteriorated to an extent we should have regarded as incredible. We are pleased to see from Miss Nutting's letter that she at least had nothing to do with the third volume: she "would have highly valued the honour of being associated" with it, but we sincerely hope that if she had been, it would have been purged of much of the vulgar self-advertisement which, as matters stand, so seriously disfigures it.—The Writer of the Review.]

* See also Hospital Institutional News this week, p. 268.—Ed. The Hospital.

